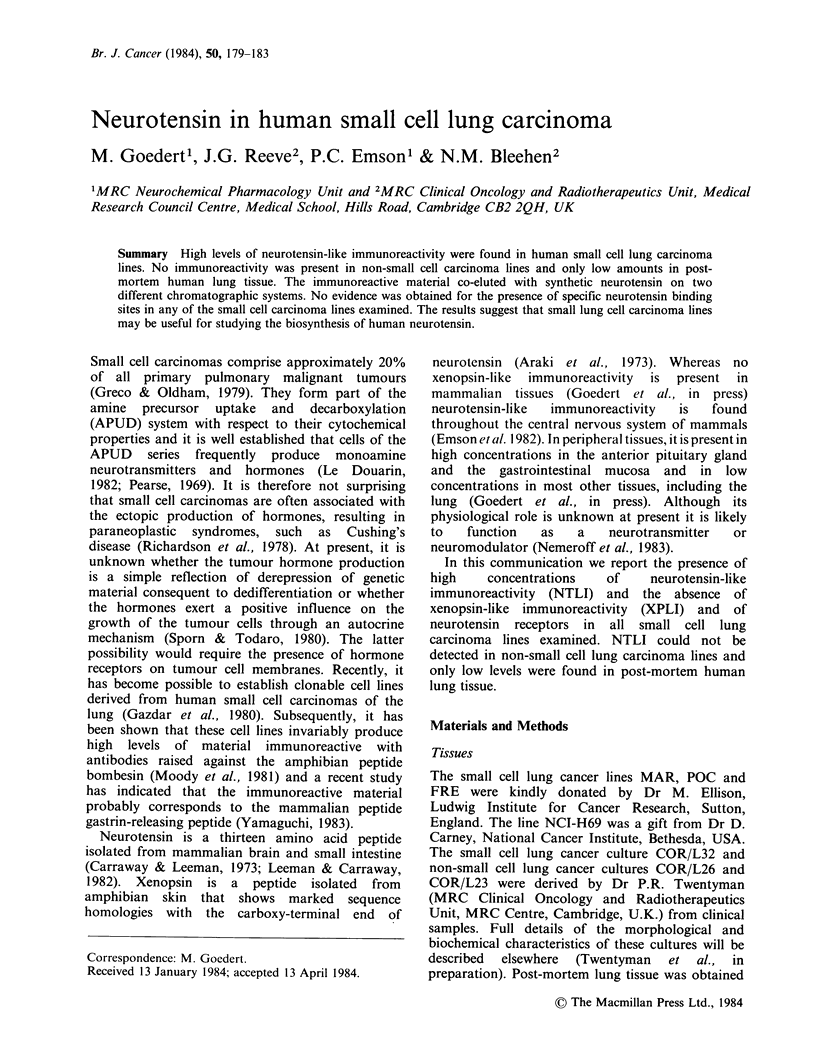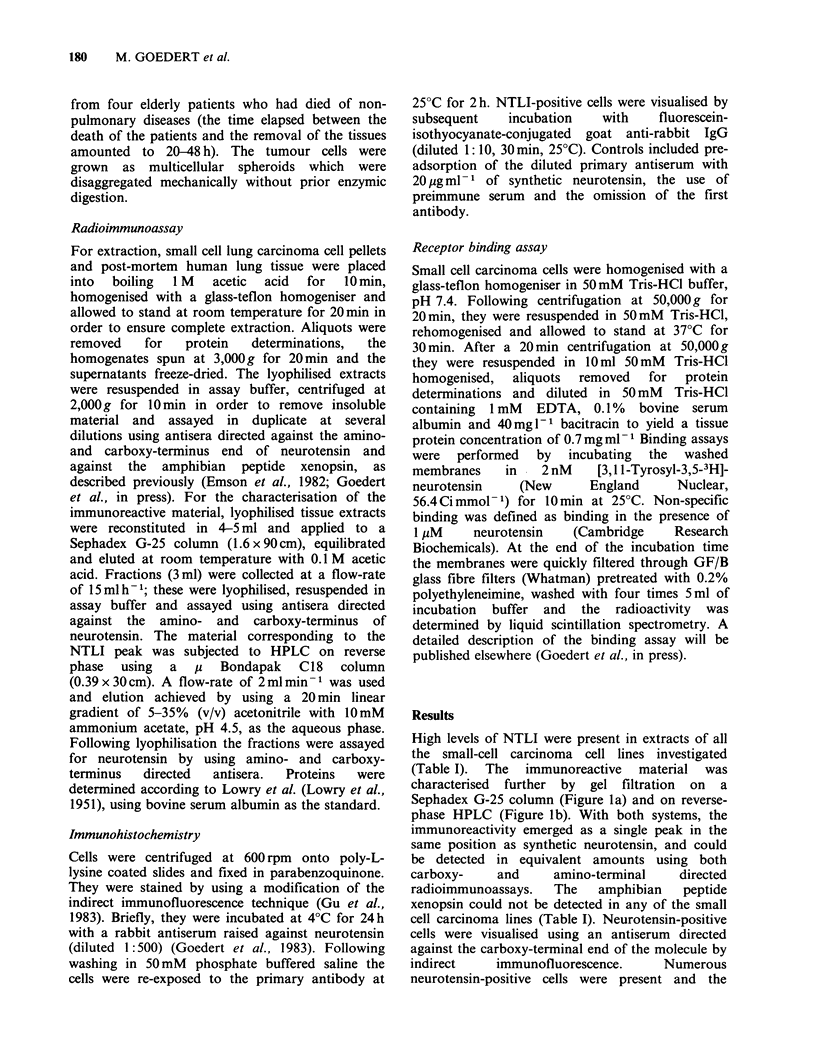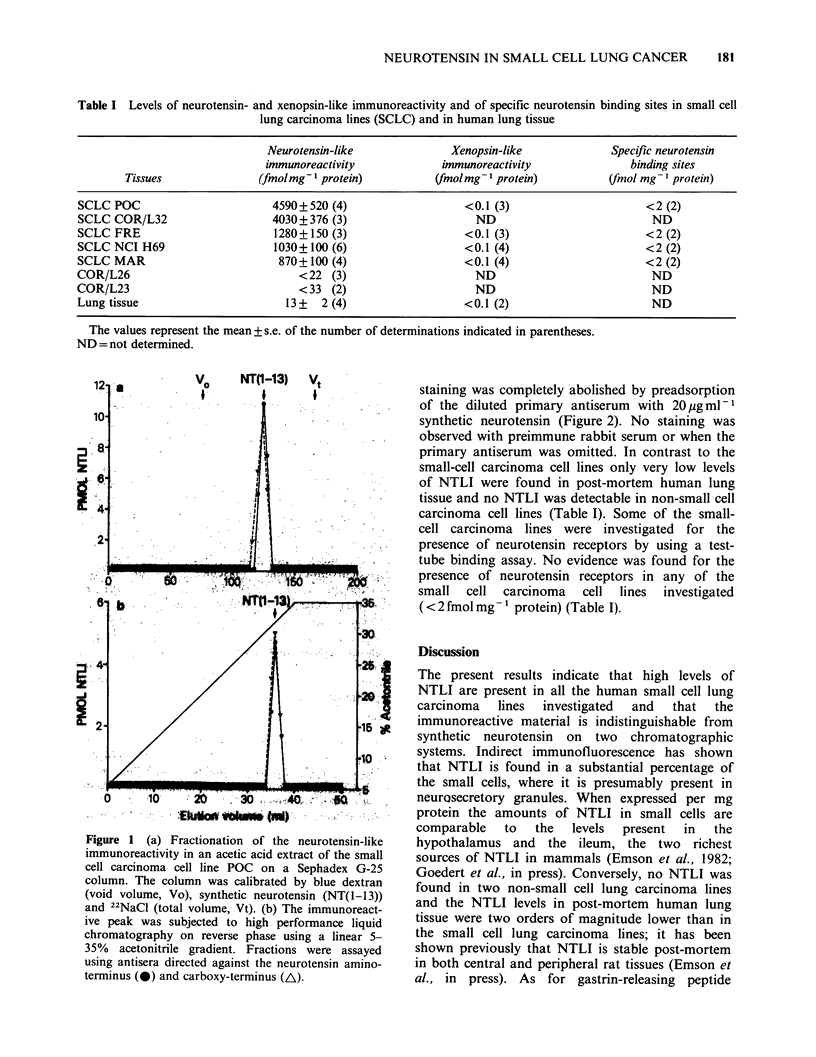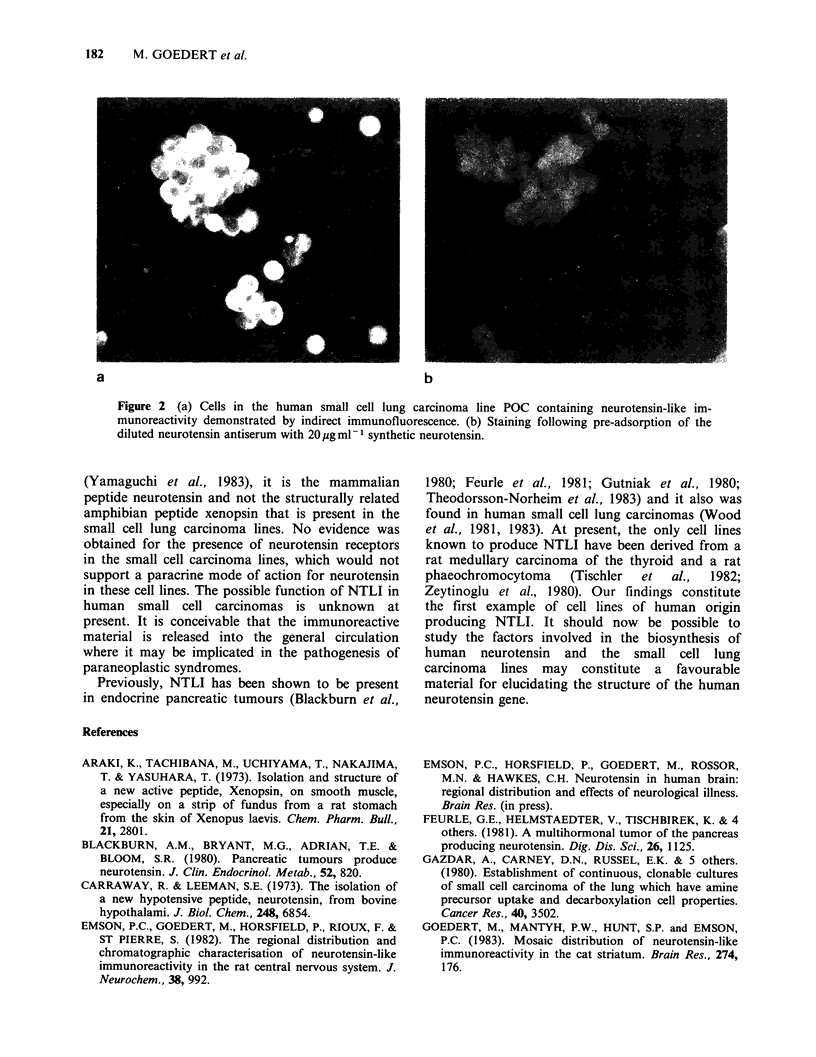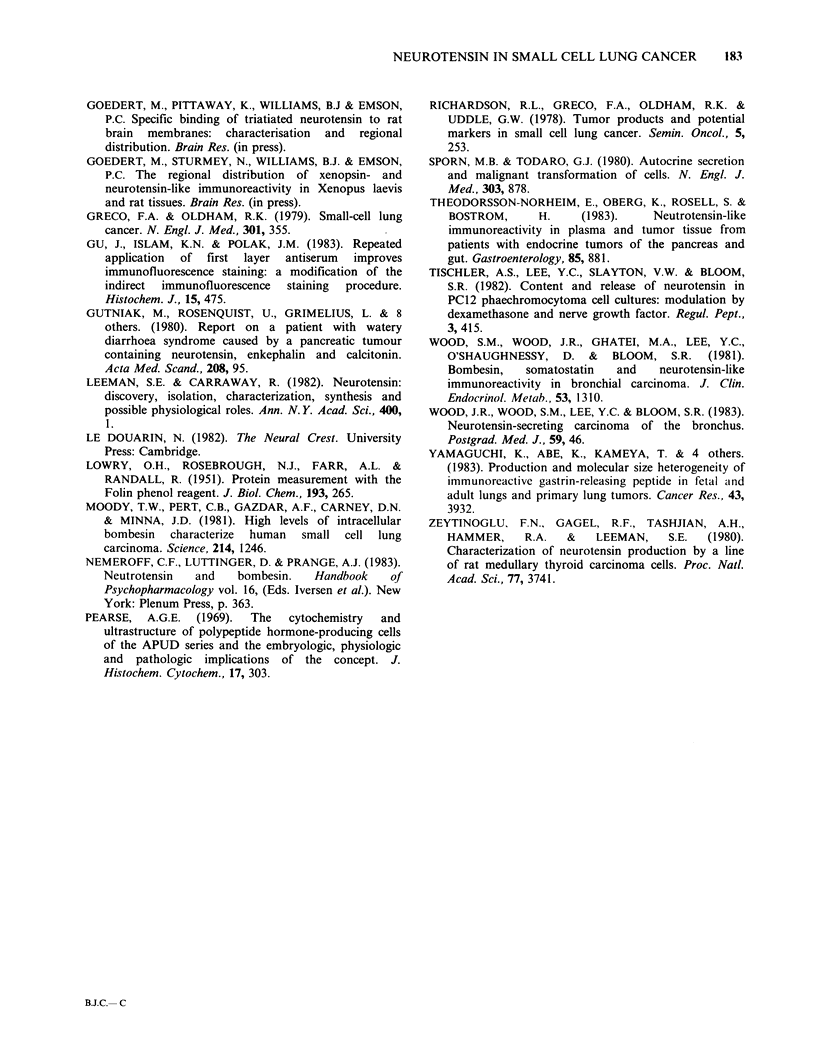# Neurotensin in human small cell lung carcinoma.

**DOI:** 10.1038/bjc.1984.160

**Published:** 1984-08

**Authors:** M. Goedert, J. G. Reeve, P. C. Emson, N. M. Bleehen

## Abstract

**Images:**


*Br. J. Cancer* (1984), **50**, 179–183

### Neurotensin in human small cell lung carcinoma

M. Goedert[1], J.G. Reeve[2], P.C. Emson[1] & N.M. Bleehen[2]

[1]*MRC Neurochemical Pharmacology Unit and* [2]*MRC Clinical Oncology and Radiotherapeutics Unit, Medical Research Council Centre, Medical School, Hills Road, Cambridge CB2 2QH, UK*

**Summary** High levels of neurotensin-like immunoreactivity were found in human small cell lung carcinoma lines. No immunoreactivity was present in non-small cell carcinoma lines and only low amounts in post-mortem human lung tissue. The immunoreactive material co-eluted with synthetic neurotensin on two different chromatographic systems. No evidence was obtained for the presence of specific neurotensin binding sites in any of the small cell carcinoma lines examined. The results suggest that small lung cell carcinoma lines may be useful for studying the biosynthesis of human neurotensin.

Small cell carcinomas comprise approximately 20% of all primary pulmonary malignant tumours (Greco & Oldham, 1979). They form part of the amine precursor uptake and decarboxylation (APUD) system with respect to their cytochemical properties and it is well established that cells of the APUD series frequently produce monoamine neurotransmitters and hormones (Le Douarin, 1982; Pearse, 1969). It is therefore not surprising that small cell carcinomas are often associated with the ectopic production of hormones, resulting in paraneoplastic syndromes, such as Cushing's disease (Richardson *et al.*, 1978). At present, it is unknown whether the tumour hormone production is a simple reflection of derepression of genetic material consequent to dedifferentiation or whether the hormones exert a positive influence on the growth of the tumour cells through an autocrine mechanism (Sporn & Todaro, 1980). The latter possibility would require the presence of hormone receptors on tumour cell membranes. Recently, it has become possible to establish clonable cell lines derived from human small cell carcinomas of the lung (Gazdar *et al.*, 1980). Subsequently, it has been shown that these cell lines invariably produce high levels of material immunoreactive with antibodies raised against the amphibian peptide bombesin (Moody *et al.*, 1981) and a recent study has indicated that the immunoreactive material probably corresponds to the mammalian peptide gastrin-releasing peptide (Yamaguchi, 1983).

Neurotensin is a thirteen amino acid peptide isolated from mammalian brain and small intestine (Carraway & Leeman, 1973; Leeman & Carraway, 1982). Xenopsin is a peptide isolated from amphibian skin that shows marked sequence homologies with the carboxy-terminal end of neurotensin (Araki *et al.*, 1973). Whereas no xenopsin-like immunoreactivity is present in mammalian tissues (Goedert *et al.*, in press) neurotensin-like immunoreactivity is found throughout the central nervous system of mammals (Emson *et al.* 1982). In peripheral tissues, it is present in high concentrations in the anterior pituitary gland and the gastrointestinal mucosa and in low concentrations in most other tissues, including the lung (Goedert *et al.*, in press). Although its physiological role is unknown at present it is likely to function as a neurotransmitter or neuromodulator (Nemeroff *et al.*, 1983).

In this communication we report the presence of high concentrations of neurotensin-like immunoreactivity (NTLI) and the absence of xenopsin-like immunoreactivity (XPLI) and of neurotensin receptors in all small cell lung carcinoma lines examined. NTLI could not be detected in non-small cell lung carcinoma lines and only low levels were found in post-mortem human lung tissue.

### Materials and Methods

### *Tissues*

The small cell lung cancer lines MAR, POC and FRE were kindly donated by Dr M. Ellison, Ludwig Institute for Cancer Research, Sutton, England. The line NCI-H69 was a gift from Dr D. Carney, National Cancer Institute, Bethesda, USA. The small cell lung cancer culture COR/L32 and non-small cell lung cancer cultures COR/L26 and COR/L23 were derived by Dr P.R. Twentyman (MRC Clinical Oncology and Radiotherapeutics Unit, MRC Centre, Cambridge, U.K.) from clinical samples. Full details of the morphological and biochemical characteristics of these cultures will be described elsewhere (Twentyman *et al.*, in preparation). Post-mortem lung tissue was obtained

Correspondence: M. Goedert.
Received 13 January 1984; accepted 13 April 1984.

© The Macmillan Press Ltd., 1984

from four elderly patients who had died of non-pulmonary diseases (the time elapsed between the death of the patients and the removal of the tissues amounted to 20–48 h). The tumour cells were grown as multicellular spheroids which were disaggregated mechanically without prior enzymic digestion.

*Radioimmunoassay*

For extraction, small cell lung carcinoma cell pellets and post-mortem human lung tissue were placed into boiling 1 M acetic acid for 10 min, homogenised with a glass-teflon homogeniser and allowed to stand at room temperature for 20 min in order to ensure complete extraction. Aliquots were removed for protein determinations, the homogenates spun at 3,000 *g* for 20 min and the supernatants freeze-dried. The lyophilised extracts were resuspended in assay buffer, centrifuged at 2,000 *g* for 10 min in order to remove insoluble material and assayed in duplicate at several dilutions using antisera directed against the amino- and carboxy-terminus end of neurotensin and against the amphibian peptide xenopsin, as described previously (Emson *et al.*, 1982; Goedert *et al.*, in press). For the characterisation of the immunoreactive material, lyophilised tissue extracts were reconstituted in 4–5 ml and applied to a Sephadex G-25 column (1.6 x 90 cm), equilibrated and eluted at room temperature with 0.1 M acetic acid. Fractions (3 ml) were collected at a flow-rate of 15 ml h$^{-1}$; these were lyophilised, resuspended in assay buffer and assayed using antisera directed against the amino- and carboxy-terminus of neurotensin. The material corresponding to the NTLI peak was subjected to HPLC on reverse phase using a μ Bondapak C18 column (0.39 x 30 cm). A flow-rate of 2 ml min$^{-1}$ was used and elution achieved by using a 20 min linear gradient of 5–35% (v/v) acetonitrile with 10 mM ammonium acetate, pH 4.5, as the aqueous phase. Following lyophilisation the fractions were assayed for neurotensin by using amino- and carboxy-terminus directed antisera. Proteins were determined according to Lowry *et al.* (Lowry *et al.*, 1951), using bovine serum albumin as the standard.

*Immunohistochemistry*

Cells were centrifuged at 600 rpm onto poly-L-lysine coated slides and fixed in parabenzoquinone. They were stained by using a modification of the indirect immunofluorescence technique (Gu *et al.*, 1983). Briefly, they were incubated at 4°C for 24 h with a rabbit antiserum raised against neurotensin (diluted 1:500) (Goedert *et al.*, 1983). Following washing in 50 mM phosphate buffered saline the cells were re-exposed to the primary antibody at 25°C for 2 h. NTLI-positive cells were visualised by subsequent incubation with fluorescein-isothyocyanate-conjugated goat anti-rabbit IgG (diluted 1:10, 30 min, 25°C). Controls included pre-adsorption of the diluted primary antiserum with 20 μg ml$^{-1}$ of synthetic neurotensin, the use of preimmune serum and the omission of the first antibody.

*Receptor binding assay*

Small cell carcinoma cells were homogenised with a glass-teflon homogeniser in 50 mM Tris-HCl buffer, pH 7.4. Following centrifugation at 50,000 *g* for 20 min, they were resuspended in 50 mM Tris-HCl, rehomogenised and allowed to stand at 37°C for 30 min. After a 20 min centrifugation at 50,000 *g* they were resuspended in 10 ml 50 mM Tris-HCl homogenised, aliquots removed for protein determinations and diluted in 50 mM Tris-HCl containing 1 mM EDTA, 0.1% bovine serum albumin and 40 mg l$^{-1}$ bacitracin to yield a tissue protein concentration of 0.7 mg ml$^{-1}$ Binding assays were performed by incubating the washed membranes in 2 nM [3,11-Tyrosyl-3,5-$^{3}$H]-neurotensin (New England Nuclear, 56.4 Ci mmol$^{-1}$) for 10 min at 25°C. Non-specific binding was defined as binding in the presence of 1 μM neurotensin (Cambridge Research Biochemicals). At the end of the incubation time the membranes were quickly filtered through GF/B glass fibre filters (Whatman) pretreated with 0.2% polyethyleneimine, washed with four times 5 ml of incubation buffer and the radioactivity was determined by liquid scintillation spectrometry. A detailed description of the binding assay will be published elsewhere (Goedert *et al.*, in press).

### Results

High levels of NTLI were present in extracts of all the small-cell carcinoma cell lines investigated (Table I). The immunoreactive material was characterised further by gel filtration on a Sephadex G-25 column (Figure 1a) and on reverse-phase HPLC (Figure 1b). With both systems, the immunoreactivity emerged as a single peak in the same position as synthetic neurotensin, and could be detected in equivalent amounts using both carboxy- and amino-terminal directed radioimmunoassays. The amphibian peptide xenopsin could not be detected in any of the small cell carcinoma lines (Table I). Neurotensin-positive cells were visualised using an antiserum directed against the carboxy-terminal end of the molecule by indirect immunofluorescence. Numerous neurotensin-positive cells were present and the

**Table I** Levels of neurotensin- and xenopsin-like immunoreactivity and of specific neurotensin binding sites in small cell lung carcinoma lines (SCLC) and in human lung tissue

| *Tissues* | *Neurotensin-like immunoreactivity (fmol mg⁻¹ protein)* | *Xenopsin-like immunoreactivity (fmol mg⁻¹ protein)* | *Specific neurotensin binding sites (fmol mg⁻¹ protein)* |
|---|---|---|---|
| SCLC POC | 4590 ± 520 (4) | <0.1 (3) | <2 (2) |
| SCLC COR/L32 | 4030 ± 376 (3) | ND | ND |
| SCLC FRE | 1280 ± 150 (3) | <0.1 (3) | <2 (2) |
| SCLC NCI H69 | 1030 ± 100 (6) | <0.1 (4) | <2 (2) |
| SCLC MAR | 870 ± 100 (4) | <0.1 (4) | <2 (2) |
| COR/L26 | <22 (3) | ND | ND |
| COR/L23 | <33 (2) | ND | ND |
| Lung tissue | 13 ± 2 (4) | <0.1 (2) | ND |

The values represent the mean ± s.e. of the number of determinations indicated in parentheses.
ND = not determined.

**Figure 1** (a) Fractionation of the neurotensin-like immunoreactivity in an acetic acid extract of the small cell carcinoma cell line POC on a Sephadex G-25 column. The column was calibrated by blue dextran (void volume, Vo), synthetic neurotensin (NT(1–13)) and $^{22}NaCl$ (total volume, Vt). (b) The immunoreactive peak was subjected to high performance liquid chromatography on reverse phase using a linear 5–35% acetonitrile gradient. Fractions were assayed using antisera directed against the neurotensin amino-terminus (●) and carboxy-terminus (△).

staining was completely abolished by preadsorption of the diluted primary antiserum with 20 $\mu g\ ml^{-1}$ synthetic neurotensin (Figure 2). No staining was observed with preimmune rabbit serum or when the primary antiserum was omitted. In contrast to the small-cell carcinoma cell lines only very low levels of NTLI were found in post-mortem human lung tissue and no NTLI was detectable in non-small cell carcinoma cell lines (Table I). Some of the small-cell carcinoma lines were investigated for the presence of neurotensin receptors by using a test-tube binding assay. No evidence was found for the presence of neurotensin receptors in any of the small cell carcinoma cell lines investigated (<2 fmol mg⁻¹ protein) (Table I).

### Discussion

The present results indicate that high levels of NTLI are present in all the human small cell lung carcinoma lines investigated and that the immunoreactive material is indistinguishable from synthetic neurotensin on two chromatographic systems. Indirect immunofluorescence has shown that NTLI is found in a substantial percentage of the small cells, where it is presumably present in neurosecretory granules. When expressed per mg protein the amounts of NTLI in small cells are comparable to the levels present in the hypothalamus and the ileum, the two richest sources of NTLI in mammals (Emson *et al.*, 1982; Goedert *et al.*, in press). Conversely, no NTLI was found in two non-small cell lung carcinoma lines and the NTLI levels in post-mortem human lung tissue were two orders of magnitude lower than in the small cell lung carcinoma lines; it has been shown previously that NTLI is stable post-mortem in both central and peripheral rat tissues (Emson *et al.*, in press). As for gastrin-releasing peptide

**Figure 2** (a) Cells in the human small cell lung carcinoma line POC containing neurotensin-like immunoreactivity demonstrated by indirect immunofluorescence. (b) Staining following pre-adsorption of the diluted neurotensin antiserum with $20\,\mu g\,ml^{-1}$ synthetic neurotensin.

(Yamaguchi *et al.*, 1983), it is the mammalian peptide neurotensin and not the structurally related amphibian peptide xenopsin that is present in the small cell lung carcinoma lines. No evidence was obtained for the presence of neurotensin receptors in the small cell carcinoma lines, which would not support a paracrine mode of action for neurotensin in these cell lines. The possible function of NTLI in human small cell carcinomas is unknown at present. It is conceivable that the immunoreactive material is released into the general circulation where it may be implicated in the pathogenesis of paraneoplastic syndromes.

Previously, NTLI has been shown to be present in endocrine pancreatic tumours (Blackburn *et al.*, 1980; Feurle *et al.*, 1981; Gutniak *et al.*, 1980; Theodorsson-Norheim *et al.*, 1983) and it also was found in human small cell lung carcinomas (Wood *et al.*, 1981, 1983). At present, the only cell lines known to produce NTLI have been derived from a rat medullary carcinoma of the thyroid and a rat phaeochromocytoma (Tischler *et al.*, 1982; Zeytinoglu *et al.*, 1980). Our findings constitute the first example of cell lines of human origin producing NTLI. It should now be possible to study the factors involved in the biosynthesis of human neurotensin and the small cell lung carcinoma lines may constitute a favourable material for elucidating the structure of the human neurotensin gene.

### References

ARAKI, K., TACHIBANA, M., UCHIYAMA, T., NAKAJIMA, T. & YASUHARA, T. (1973). Isolation and structure of a new active peptide, Xenopsin, on smooth muscle, especially on a strip of fundus from a rat stomach from the skin of Xenopus laevis. *Chem. Pharm. Bull.*, **21,** 2801.

BLACKBURN, A.M., BRYANT, M.G., ADRIAN, T.E. & BLOOM, S.R. (1980). Pancreatic tumours produce neurotensin. *J. Clin. Endocrinol. Metab.*, **52,** 820.

CARRAWAY, R. & LEEMAN, S.E. (1973). The isolation of a new hypotensive peptide, neurotensin, from bovine hypothalami. *J. Biol. Chem.*, **248,** 6854.

EMSON, P.C., GOEDERT, M., HORSFIELD, P., RIOUX, F. & ST PIERRE, S. (1982). The regional distribution and chromatographic characterisation of neurotensin-like immunoreactivity in the rat central nervous system. *J. Neurochem.*, **38,** 992.

EMSON, P.C., HORSFIELD, P., GOEDERT, M., ROSSOR, M.N. & HAWKES, C.H. Neurotensin in human brain: regional distribution and effects of neurological illness. *Brain Res.* (in press).

FEURLE, G.E., HELMSTAEDTER, V., TISCHBIREK, K. & 4 others. (1981). A multihormonal tumor of the pancreas producing neurotensin. *Dig. Dis. Sci.*, **26,** 1125.

GAZDAR, A., CARNEY, D.N., RUSSEL, E.K. & 5 others. (1980). Establishment of continuous, clonable cultures of small cell carcinoma of the lung which have amine precursor uptake and decarboxylation cell properties. *Cancer Res.*, **40,** 3502.

GOEDERT, M., MANTYH, P.W., HUNT, S.P. and EMSON, P.C. (1983). Mosaic distribution of neurotensin-like immunoreactivity in the cat striatum. *Brain Res.*, **274,** 176.

GOEDERT, M., PITTAWAY, K., WILLIAMS, B.J & EMSON, P.C. Specific binding of triatiated neurotensin to rat brain membranes: characterisation and regional distribution. *Brain Res.* (in press).

GOEDERT, M., STURMEY, N., WILLIAMS, B.J. & EMSON, P.C. The regional distribution of xenopsin- and neurotensin-like immunoreactivity in Xenopus laevis and rat tissues. *Brain Res.* (in press).

GRECO, F.A. & OLDHAM, R.K. (1979). Small-cell lung cancer. *N. Engl. J. Med.*, **301**, 355.

GU, J., ISLAM, K.N. & POLAK, J.M. (1983). Repeated application of first layer antiserum improves immunofluorescence staining: a modification of the indirect immunofluorescence staining procedure. *Histochem. J.*, **15**, 475.

GUTNIAK, M., ROSENQUIST, U., GRIMELIUS, L. & 8 others. (1980). Report on a patient with watery diarrhoea syndrome caused by a pancreatic tumour containing neurotensin, enkephalin and calcitonin. *Acta Med. Scand.*, **208**, 95.

LEEMAN, S.E. & CARRAWAY, R. (1982). Neurotensin: discovery, isolation, characterization, synthesis and possible physiological roles. *Ann. N.Y. Acad. Sci.*, **400**, 1.

LE DOUARIN, N. (1982). *The Neural Crest.* University Press: Cambridge.

LOWRY, O.H., ROSEBROUGH, N.J., FARR, A.L. & RANDALL, R. (1951). Protein measurement with the Folin phenol reagent. *J. Biol. Chem.*, **193**, 265.

MOODY, T.W., PERT, C.B., GAZDAR, A.F., CARNEY, D.N. & MINNA, J.D. (1981). High levels of intracellular bombesin characterize human small cell lung carcinoma. *Science*, **214**, 1246.

NEMEROFF, C.F., LUTTINGER, D. & PRANGE, A.J. (1983). Neutrotensin and bombesin. *Handbook of Psychopharmacology* vol. 16, (Eds. Iversen *et al.*). New York: Plenum Press, p. 363.

PEARSE, A.G.E. (1969). The cytochemistry and ultrastructure of polypeptide hormone-producing cells of the APUD series and the embryologic, physiologic and pathologic implications of the concept. *J. Histochem. Cytochem.*, **17**, 303.

RICHARDSON, R.L., GRECO, F.A., OLDHAM, R.K. & UDDLE, G.W. (1978). Tumor products and potential markers in small cell lung cancer. *Semin. Oncol.*, **5**, 253.

SPORN, M.B. & TODARO, G.J. (1980). Autocrine secretion and malignant transformation of cells. *N. Engl. J. Med.*, **303**, 878.

THEODORSSON-NORHEIM, E., OBERG, K., ROSELL, S. & BOSTROM, H. (1983). Neutrotensin-like immunoreactivity in plasma and tumor tissue from patients with endocrine tumors of the pancreas and gut. *Gastroenterology*, **85**, 881.

TISCHLER, A.S., LEE, Y.C., SLAYTON, V.W. & BLOOM, S.R. (1982). Content and release of neurotensin in PC12 phaechromocytoma cell cultures: modulation by dexamethasone and nerve growth factor. *Regul. Pept.*, **3**, 415.

WOOD, S.M., WOOD, J.R., GHATEI, M.A., LEE, Y.C., O'SHAUGHNESSY, D. & BLOOM, S.R. (1981). Bombesin, somatostatin and neurotensin-like immunoreactivity in bronchial carcinoma. *J. Clin. Endocrinol. Metab.*, **53**, 1310.

WOOD, J.R., WOOD, S.M., LEE, Y.C. & BLOOM, S.R. (1983). Neurotensin-secreting carcinoma of the bronchus. *Postgrad. Med. J.*, **59**, 46.

YAMAGUCHI, K., ABE, K., KAMEYA, T. & 4 others. (1983). Production and molecular size heterogeneity of immunoreactive gastrin-releasing peptide in fetal and adult lungs and primary lung tumors. *Cancer Res.*, **43**, 3932.

ZEYTINOGLU, F.N., GAGEL, R.F., TASHJIAN, A.H., HAMMER, R.A. & LEEMAN, S.E. (1980). Characterization of neurotensin production by a line of rat medullary thyroid carcinoma cells. *Proc. Natl. Acad. Sci.*, **77**, 3741.